# A Review of Insect Pest Management in Vegetable Crop Production in Nigeria

**DOI:** 10.3390/insects14020111

**Published:** 2023-01-21

**Authors:** Thomas I. Ofuya, Adeyela I. Okunlola, George N. Mbata

**Affiliations:** 1 Department of Crop, Soil & Pest Management, The Federal University of Technology, Akure PMB 704, Nigeria; 2 Agricultural Research Station, Fort Valley State University, 1005 State University Drive, Fort Valley, GA 31030, USA

**Keywords:** biopesticides, chemical control, insect pests, IPM, natural enemies, vegetable and fruit crops, Nigeria

## Abstract

**Simple Summary:**

Vegetable crops are grown in Nigeria for their edible fruits, leaves, and succulent shoots and roots. The vegetables include okra, tomatoes, chilli peppers, cucumbers, green amaranth, carrots and onions. Insect pest infestations and damages to these crops can greatly limit their availability for human consumption if unchecked. The insect pests include foliage beetles, caterpillars, aphids, fruit flies, stink bugs, and grasshoppers. Vegetable growers have many different chemical and non-chemical methods that can be used to mitigate insect damage to vegetable crops including the application of synthetic insecticides, modification of cultural practices, use of resistant varieties, application of botanicals and conservation practices for natural enemies of the insect pests, and these are presented in this review. By combining as many pest management tools as practicable, the insect pest problems of vegetables in Nigeria can be reasonably managed in a sustainable manner.

**Abstract:**

Insect pest infestations and damage can limit the production of vegetables in the farming systems in Nigeria. This review looks at integrated insect pest management as a possible panacea for resolving insect pest issues in vegetable crops. The main vegetable crops which include okra, tomatoes, chilli peppers, cucumbers, green amaranth, carrots and onions are highlighted. The major insect pests of the various vegetables which include foliage beetles, caterpillars, aphids, fruit flies, stink bugs, and grasshoppers are also mentioned. The various control measures that have been empirically verified for the mitigation of the impact of these insect pests, including the application of synthetic insecticides, modification of agronomic practices, use of resistant varieties, application of botanicals, biological and mechanical controls, are discussed. Studies which have been carried out attempting to integrate two or more of the control strategies for better insect pest control are also reviewed. Strategies that can be put in place for the integrated pest management of vegetable insect pests in Nigeria are considered. Among the IPM (Integrated Pest Management) practices instituted for the mitigation of pest infestations on vegetable crops in Nigeria, intercropping of suitable vegetables in combination with the application of aqueous extracts of *Azadirachta indica* and *Piper guineense* seeds under good farm hygiene and sanitation proved to be most successful.

## 1. Introduction

Insect pests are the major causes of crop yield losses around the world and pest management plays a critical role in providing food security and farming income [1]. Nigel [2] estimated that global losses in vegetable production amounts to 27.7% of which 8.7% is attributable to insect pests that have the potential to cause more losses if unchecked. The Food and Agriculture Organisation (FAO) [3] also reported that the estimated annual global losses of vegetables due to insects alone stands at about 15–20% during field production and 18–20% during storage. Damage by insect pests is certainly one of the main limiting factors to increased production of vegetables in farming systems in Nigeria and constitutes the primary cause of low-quality and poor yields [4]. For instance, the flea beetles, *Podagrica* spp. can reduce okra (*Abelmoschus esculentus* Moench) in Nigeria by as much as 45% [5]. About an 80–100% yield loss in tomato has been recorded in Nigeria due to damage caused by the leaf miner, *Tuta absoluta* (Meyrick) [6]. The losses attributable to insect pests undoubtedly have the potential to be aggravated if unchecked, especially with dramatic climate events such as unusual rainfalls or severe drought, which may result in increased pest populations.

Many chemical and non-chemical control options are available to vegetable producer in Nigeria for dealing with insect pest problems, including the modification of cultural practices, use of resistant varieties, application of synthetic insecticides, application of botanicals and conservation practices for natural enemies of the insect pests. Integrated pest management (IPM) simply signifies the application of the best mix of environmentally sound techniques in order to keep pests below numbers that can cause unacceptable damage. In practice, IPM means avoiding the dependence on one single method for controlling pests; various measures are undertaken, reinforcing one another, to ensure that pest populations remain below that which will cause unacceptable damage. The ultimate objective is to reduce the dependence on chemical control through the use of synthetic pesticides as the main input for crop pest management. Reduction in the use of pesticides lessens environmental pollution and minimizes the risks of poisoning the user and consumer alike.

When insect pest population densities exceed a pre-established or conceptualized threshold capable of causing economic loss [7], a mitigation tool must be instituted to reduce damage. This should form the basis for decisions in IPM programs. Economic injury and threshold levels are yet to be determined for the majority of vegetable insect pests in Nigeria. Amongst the few determined, the maize moth, *P. bipunctalis,* where the calculated threshold level for pesticide application is three larvae per plant, the melon fly, *D. cucurbitae* where the recommended economic threshold is 10% fruit infestation, and the cabbage aphid, *B. brassicae,* where a threshold of five aphids per plant on two-week old plants requires control [8]. Chemical insect pest control measures are usually applied to vegetable crops in Nigeria pre-emptively without considering the economic levels. This is understandable because vegetable producers are aware of the insect pest problems which may be severe if unchecked.

In this review the main vegetable crops grown in Nigeria are outlined as well as their major insect pests, and the various control measures that have been empirically verified for the management of these pests. Studies which have been carried out attempting to integrate two or more of the control strategies for better pest management are also reviewed. Strategies that can be put in place for the integrated pest management of vegetable insect pests in Nigeria are finally suggested.

## 2. Vegetable Crops in Nigeria

Vegetable crops are cultivated abundantly in Nigeria among farming communities. The commonly consumed vegetables include those cultivated for their edible fruits (fruit vegetables): *Trichosanthes cucumerine* L. (snake gourd), *Abelmoschus esculentus* (L.) Moench (okra), *Solanum melongena* L. (eggplant or garden egg), *Capsicum frutescens* L. (chilli pepper), *Capsicum annum* L. (sweet chilli pepper), *Lycopersicon esculentum* L. (tomato), *Cucumis sativus* L. (cucumber), *Citrullus lunatus* (Thumb.) (watermelon) and *Citrullus colocynthis* (L.) Schrad (egusi melon). Vegetables grown for their edible leaves and succulent shoots (leaf vegetables) include *Amaranthus hybridus* L. (green amaranth), *Celosia argentea* L. (plumed cockscomb or silver cockscomb), *Cucurbita maxima* Duchesne (squash), *Cucurbita pepo* L. (field pumpkin) *Telfaria occidentalis* Hook.f. (fluted pumpkin), *Hibiscus sabdariffa* L. (roselle), *Corchorus olitorius* L. (Jew’s mallow), *Basella alba* L. (Indian spinach), *Crassocephalum biafrae* S. Moore (worow), *Talinum triangulare* (Jacq.) Willd. (Water leaf) and *Vernonia amygdalina* Delite (bitter leaf). *Brassica oleracea* L. (cabbage) and *Lactuca sativa* L. (lettuce) are introduced leafy vegetables that are increasingly being cultivated in West Africa [9]. Root vegetables include *Daucus carota* (Hoffm.) (carrot), *Allium cepa* L. (onion), *Allium sativum* L. (garlic), *Zingiber officinale* Roscoe (ginger) and *Curcuma longa* L. (turmeric). 

Nigerian vegetable output in 2020 was 15.7 million tons [10] which includes 3.7, 1.8, 1.4, 1.0, and 0.7 million tons of tomatoes, okra, onions, cucumber and ginger, respectively. Small holder farmers make up the larger percentage of vegetable producers in Nigeria and the crops are frequently intercropped with cereals, legumes and root and tuber crops, with relatively low production technology [11]. Vegetables are very important sources of vitamins and minerals, and other protective nutrients for human health [12]. In traditional farming systems, vegetables are easily grown. Gains that may be accruable to a thriving vegetable production in Nigeria have been suggested to include eradication of extreme hunger and poverty as a result of higher incomes and stronger rural economies, promotion of healthy living, ensuring environmental sustainability, job creation and improved food and nutrition security [12,13]. Thriving vegetable production in Nigeria may increase government revenue through the export of produce, such as peppers, ginger and turmeric.

## 3. Insect Pests of Vegetable Crops

Akinlosotu [14] listed 155 insect species from 44 families and seven orders that caused varying degrees of damage to vegetables in southwestern Nigeria. Ogbalu et al. [15] also identified 40 insect species from six orders and 19 families as destructive pests of vegetables. More recently, Aderolu et al. [4] provided another comprehensive listing of insects associated with amaranths in Ibadan, southwestern Nigeria consisting of sixty species from 29 families and 12 orders; comprising 31 defoliators, 12 predators, one pupal parasitoid (*Apanteles hymeneae*) and 16 non-economic species. The predators are mainly coccinellids, such as *Cheilomenes* spp., and reduviids, such as *Rhynocoris* spp. Many other studies have reported and confirmed the different insect pest species of vegetable crops from different geographical areas of Nigeria [8,16,17,18,19,20,21,22,23,24,25,26,27,28,29]. The major insect pests of some vegetables crops in Nigeria are presented in Table 1.

## 4. Management of Insect Pests of Vegetable Crops

Chemical and non-chemical control methods are traditionally used by farmers for the control of vegetable crop insect pests in Nigeria. Chemical control encompasses the use of synthetic insecticides while non-chemical control includes methods that do not involve the use of synthetic insecticides. This encompasses cultural, mechanical and biological control as well as the use of plant-derived insecticides (botanicals) that can be prepared by farmers from local products. Pest mitigation practices and results in Nigeria in this regard are discussed.

### 4.1. Application of Synthetic Insecticides 

Growers of vegetables in Nigeria and West Africa in general use a wide range of insecticides for the control of insect pests and increased yield [9,32]. Many of the insecticides used for vegetable cultivation in Nigeria have a contact mode of action and are lethal to specific insects once absorbed via contact; for example, dimethoate, carbaryl, cypermethrin, deltamethrin, and lambda cyhalothrin. Other insecticides applied to protect vegetables have systemic action and are soluble in water, resulting in being absorbed and circulated in plant tissues where they can be picked up by insects [8,33,34,35,36,37,38,39,40,41,42]. Example of systemic pesticides used in Nigeria include neonicotinoids/nitroguanidine (imidacloprid) and dimethoate (locally systemic). The effectiveness of these synthetic chemicals in the control of vegetable insect pests has been reported by several workers [8,33,34,35,36,37,38,39,40,41] (Table 2). Umeh et al. [18] noted that inappropriate application of insecticides and the use of wrong dosages resulted in insect control failures in tomato production. Benson et al. [43] showed that the application of synthetic insecticides in the production of okra in southwestern Nigeria may be profitable based on a gross margin and benefit cost analysis. Nevertheless, the use of synthetic insecticides on vegetables is not encouraged due to the health and environmental concerns. Synthetic chemicals are hazardous to humans, consumers and handlers alike, and this is particularly critical in Nigeria where pesticide education is low, and the tendency of misuse is very high. More worrisome is the fact that many of the synthetic chemicals which have been banned for use in many countries are still being used in West Africa [19]. Other drawbacks in the use of synthetic insecticides for the management of insect pests include toxicity to beneficial arthropods, such as pollinators, parasitoids and predators. Dependency on pesticide application has resulted in the development of pesticide resistance in pests, prohibitive costs and inconsistent supplies. The sole dependence on the use of insecticides by farmers without regard to IPM, resulted in the widespread high levels of resistance by *T. absoluta* to chlorantraniliprole + lamda-cyalothrin in Nigeria [44]. This underscores the need to seek alternative pest management tools that are compatible with the environment to replace these synthetic chemicals.

### 4.2. Cultural Control

Adopting appropriate cultural agronomic practices that prevent or reduce insect pest infestations has been passed on from generation to generation by Nigerian farmers. Cultural practices are the least expensive options available to farmers. The following practices are considered:

#### 4.2.1. Land Preparation

Land preparation practices for vegetable planting in the field, such as bush clearing, ploughing, harrowing, ridging, etc., carried out by farmers may have pest management implications. Soil tillage and prompt destruction of crop residues have been suggested to reduce the incidence of lepidopterous as well as some other insect pests of vegetables that over-season on crop remains on the field [8]. Soil tillage and flooding have been demonstrated to mitigate the incidence of the grasshopper, *Zonocerus variegatus* L. a general insect pest of vegetables in Nigeria [45]. Soil tillage and subsequent exposure to desiccation destroys the eggs of pests, while flooding delays egg development and eclosion [45]. Two possibilities are open to vegetable farmers to use, including soil tillage and flooding, for *Z. variegatus* control. 

#### 4.2.2. Time of Planting and Plant Population Manipulation

Time of planting and crop density can be manipulated in vegetable production for insect pest control purposes. Varying the planting time of crops works as a means of cultural control by creating asynchrony between crop phenology and that of the pest; thus, retarding the rate of colonization or it may mean that the pest fails to coincide with a critical growth stage [7]. For instance, Oke and Ofuya [46] reported that the population of the plant bug *Cletus fuscescens* Walker fluctuated relative to overall phenological stage of amaranth vegetable crop, with the peak population coinciding with the grain milky seed stage; suggesting that early planting in the season will reduce infestation and damage by the bug. Increased plant density has been reported to reduce the number of carrot-willow aphid, *Cavariella aegopodii* Scop. infestations of carrots in the field [31]. Observation of a closed season is frequently recommended for the management of some insect pests of vegetable crops as well as other crops in Nigeria, such as Spiny Bollworm, *Earias insulana* Boisduval, a major pest of cotton but will also attack okra, kenaf and roselle [8]. A closed season is when the sowing of a new cotton crop is delayed for at least two months after the harvest of the previous crop. Growing of alternative host plants, such as okra or roselle, should be avoided during the closed season. The planting of the preferred host in one region should all be concentrated in one season so that at other times a closed season can be observed [8].

#### 4.2.3. Supplemental Application of Soil Nutrients

Vegetable farmers use organic and mineral fertilizers to help soils to recover from nutrient losses, and in some cases reduce pest problems [9]. However, some studies have shown that the application of fertilizers can intensify the pest colonization of crops which was, for instance, observed for musk pumpkin fertilized with organic manure and NPK [47]. It is therefore imperative and best to determine the level of fertilizer application that encourages natural enemies and optimizes yield.

#### 4.2.4. Intercropping

Farmers frequently intercrop vegetables on the farm and a single bed can hold as many as five different vegetables or crops [9]. Crops intercropped with tomato in the northern parts of Nigeria may include lettuce, cabbage, peas, carrot, onions and peppers while in the south the crops include okra, peppers, amaranths, *Celosia* spp., *Corchorus* spp., maize, cassava and yam [18]. Intercropping has been investigated for the management of insect pests of vegetable crops. For instance, Uvah and Coaker [31] reported that intercropping of carrots with onions reduced attacks by carrot fly, *Psila rosae* Fab. and *Thrips tabaci* Lind., respectively, compared with those on carrots and onions in monoculture. It was further observed that carabid and staphylinid predators of *P. rosae* were more abundant on intercropped plots which may partly explain the reduced attack by the pests. Okunlola [48] also reported that growing three leafy vegetables: *A. hybridus*, *C. argentia* and *C. olitorius* in mixed cropping systems significantly reduced the insect pest incidence and damage to the crops in comparison with the sole crops. There is a report that a leaf beetle infestation on okra grown at high densities can be reduced by intercropping with legume crops (e.g., beans) [49]. Zakka et al. [50] further reported the effectiveness of maize (*Zea mays* L.) as an intercrop in the management of insect pests of okra. Okra intercropped with maize in the ratio of 1:1 was observed to be most effective in reducing insect infestation of okra and may need to be promoted. Intercropping okra with *Amaranthus* may help to reduce pest populations, such as, *Podagrica* spp. [8]. Intercropping peppers with taller crops such as maize or sorghum can reduce infestations by *M. persicae* presumably by the taller crop acting as a physical barrier to aphid invasion [8]. Alternate intercropping of chilli pepper and cowpea has also been demonstrated to have pest management and yield benefits for both crops [51]. Intercropping ginger with okra significantly decreased populations of *B. tabaci* and *P. sjostedti* attacking the okra plants and resulted in significant fruit yield in comparison with the monocropping of okra. Fruit damage in okra by insects such as *Podagrica* spp. and *D. supersitiosus* in an okra/pepper intercropping study was reduced by 50% in comparison to damage in the sole cropping systems [52].

#### 4.2.5. Weed Control

Weeds that are closely related to cultivated crops may harbour insects that may infest and damage such crops [53], and the removal of such weeds may be helpful in insect pest control. Weeds have been observed to influence the colonization of some crops by field insect pests in Nigeria, and therefore can be manipulated for pest management purposes [54,55,56]. Weeds, such as *Euphorbia hirta* L., E. *glomifera* (Millsp) DC and *Boerhaavia diffusa* L., serve as alternative hosts of *Aphis craccicora* Koch, and their elimination in the vicinity of farms can reduce infestations of leguminous crops [57]. However, the relationship between weed removal regimes and insect pest infestation needs to be experimentally verified for different vegetable crops.

#### 4.2.6. Crop Rotation

Crop rotation enables farmers to subject their farm to continuous cultivation by planting different crops in successive seasons. In many parts of Nigeria, farmers for example rotate tomato with other crops, such as cereals, tubers, and other vegetables [18]. Crop rotation helps to control insect pest populations by interfering with the life cycle if the rotation crop is a non-host of the pest to be regulated. It is possible to control some specific injurious insects of vegetables by carefully rotating different leafy, fruit, and root vegetables on the same farms but this should be subjected to empirical verification. The practice of suitable crop rotation has been suggested as a non-chemical method for the control of cabbage aphid, *B. oleracea* [8].

#### 4.2.7. Trap Cropping

A trap crop is a plant specifically grown to attract an insect pest away from another more economic or primarily desired crop [7]. Trap crops may work particularly well at smaller production scales, being highly amenable where crop diversification and a reduction of synthetic inputs are prioritised over yield alone [8]. There is an indication that trap crops or plants may reduce infestations of okra by some insect pests [58], where roselle and cowpea plants trapped leafhopper, cotton-stainer and flea beetle pests of okra. The use of trap crops may therefore be of potential value in small farm holdings of vegetable farmers in Nigeria but needs to be further explored and exploited. The trap cropping concept partly plays out in the use of intercropping for pest management.

#### 4.2.8. Use of Plant-Derived Insecticides or Botanicals

Plant-derived insecticidal formulations, such as aqueous wood ash solution, are widely used by subsistence and transitional farmers in low-income countries [18,59]. They are used because they are putatively biodegradable, have low mammalian toxicity, safe to natural enemies of pests, cheap and can be produced by skilled and unskilled farmers [47], but some of these claims are yet to be adequately proven. 

Many studies have shown unequivocally that aqueous extracts of many plants when sprayed on cultivated vegetables can reduce insect pest infestation and increase yield (Table 3). 

In most cases, when the plant materials are applied singly, they are less effective in insect pest control in comparison with synthetic insecticides. However, when different plant materials are combined in aqueous formulation, they are sometimes as effective as the synthetic chemicals in the control of vegetable insect pests. For example, Benson et al. [43] reported that the mixture of aqueous extracts of *Azadirachta indica* and *Piper guineense* were effective and profitable in the management of insect pests of okra, such as flea beetles, in Nigeria and can be used to replace the synthetic chemicals carbaryl and lambda-cyhalothrin which did not surpass in efficacy. The efficacy of some aqueous plant extracts against field pests of vegetables (e.g., flea beetles on okra) has further been verified under laboratory conditions [69,71]. It is suggested that insecticidal aqueous cocktails or formulations containing products from three or more plants need to also be tested for their efficacy in controlling infestation and damage by insect pests to vegetable crops because they may prove more efficacious than the synthetic insecticides. It is important that cognizance is taken of the factors that may affect the efficacy of botanicals, such as variations in the quantity of plant material and consequently active ingredients as well as variations in the preparation of formulation process. There is the need for standardization in the preparation and formulation of these botanical insecticides.

#### 4.2.9. Use of Insect Resistant Vegetable Varieties

Growing varieties of vegetable crops with complete or even partial resistance to pest insects can be an effective way of reducing crop damage. Studies in Nigeria have shown that the majority of farmers are willing to accept cultivation of pest-resistant crop genotypes [72]. It has been indicated that host plant resistance should form the foundation for integrated pest management [73]. However, breeding for insect tolerance or resistance in vegetable crops has not been adequately explored in Nigeria. Oke et al. [74] observed that the level of resistance to insect pests in cultivated amaranth (*Amaranthus cruentus* L.) is low, but tested germplasm exhibited a variability of resistance to the bug *Cletus fuscescens* Walker infestations along with accessions with unique seed viability percentage and seed yield that could be explored to develop high yielding grain amaranth varieties with tolerance or resistance to the pest. Oke et al. [75] also reported that inflorescence density and morphology in grain amaranth was a factor in the low susceptibility of the vegetable to *C*. *fuscescens* and concluded that dense inflorescence in amaranth accessions could be a source for breeding programmes for conferment of antixenosis resistance to *C. fuscescens* attacks.

Crops can also be modified to resist insect pest attacks through genetic manipulations leading to the production of genetically modified varieties, but this has not yet been carried out with vegetable crops in Nigeria. However, genetically modified cowpea, *Vigna unguiculata* (L.) Walpers with resistance to the devastating cowpea pod borer *Maruca vitrata* Fabricius has been produced in Nigeria and has been distributed to farmers for the cultivation and enhanced production of this protein-rich legume [76].

### 4.3. Mechanical Control

Mechanical insect pest control is the management of insect pest populations using physical means, such as manual removal, use of surface irrigation to dislodge pests, use of traps or barriers to exclude pests, etc. Vegetable growers in Nigeria carry out some mechanical measures to mitigate insect pest infestations and damage. These include the hand-picking of insects, such as *Z. variegatus* and lepidopterous caterpillars, and the removal of foci of infestations which could be leaves for *S. litoralis* in onions, and hand washing of cabbage to alleviate *B. brassicae* [8]. For the leaf roller, *S. derogata* that attacks okra, eggplant, tomato and others, larvae feed inside a shelter formed from the rolled leaves which also gives them protection against contact insecticides. Rolled leaves can be picked by hand and destroyed [8]. These measures are effective at low and early infestations. It is recommended that after harvesting vegetable crops, such as okra, tomato, peppers, etc., plants should be uprooted and destroyed or ploughed under the soil [8]. Fruit fly traps are effective in the control of melon fly, *D. cucurbitae* [7]. These are made from containers with narrow openings, such as a funnel, which allow the flies to enter freely but restrict their exit. The containers can be baited with a substance that the flies find attractive, such as pumpkin juice, peptone, honey, and cooking oil [77].

### 4.4. Biological Control

The insect fauna of crop agro-ecosystems in Nigeria frequently include insect species which serve as natural enemies, such as parasitoids and predators of the insect pests [78]. Aderolu et al. [4] observed 12 predators and one pupal parasitoid, Apanteles hymenaea (Hymenoptera: Braconidae). Saethre et al. [79] reported the natural enemies of *Aphis gossypii* Glover that infest a wide range of vegetables in the West African sub-region to include predators especially *Cheilomenes* spp. and *Ischiodon aegyptus* (Wieldermann), the obligate entomopathogen *Neozygites* spp., the parasitoids *Lysiphlebus testaceipes* (Cressan) and *Aphelinus ficusae* (Prinsloo and Neser) as well as hyperparasitoids especially *Syrphophagous africanus* (Gahan). Reduviids, especially *Rhynocorus* spp. are important predators of many insect pests of vegetable pests such as *Podagrica* spp., *Dysdercus* spp., *Helicoverpa* spp., *Spodoptera* spp. and *Mylabris* spp. [80]. Intercropping has been observed to promote the activities of natural enemies in vegetable crops in Nigeria, such as carabid and staphylinid, predators of *P. rosae* in carrot and onion mixed cropping [31]. 

Use of entomopathogens, (organisms that can cause disease and death in insects but not in vertebrates), is an integral part of biological control. These organisms include nematodes and fungi. The use of entomopathogens has not been adequately researched for vegetable insect pest management in Nigeria [81]. Bob-Manuel [82] demonstrated the effective dissemination and persistence of the disease caused by the entomopathogenic fungus *Hirsutella thompsonii* Fisher on the cassava green mite, *Mononychellus tanajoa* Bondar in Nigeria. Elsewhere, in the complex of biological control agents, entomopathogenic fungi appear to be more effective and most successfully utilized insect pathogen, especially in the genera *Aspergillus*, *Beauvaria*, *Metarhizium* and *Lecanicillium* [83]. Preliminary work by Ottun et al., [84] revealed that there is great potentialfor the possibility of using entomopathogenic nematodes in the management of insect pests in Nigeria. 

More critical studies need to be undertaken in Nigeria to determine the impact of the natural enemies of the different insect pests of vegetables in their population regulation. Undoubtedly, provisions should be made for the regulation of vegetable insect pest populations by natural enemies. Thus, practices that encourage and conserve the activities of the natural enemies should form components of the integrated pest management system for vegetable crops.

### 4.5. Integrated Pests Management in Vegetable Crops

Suleiman [24] reported that some vegetable farmers in Nigeria already use a combination of methods, such as chemical and cultural, chemicals and botanicals, etc., in controlling pests of vegetables. Okunlola and Ofuya [85] reported that mixed cropping involving *C. olitorius* L., *A. hybridus* and *C. argentea* and the plant extract sprays of *A. indica* and *P. guineense* interacted positively to control insect pests and enhance the yields of these vegetables. Aderolu et al. [4] recommended the use of pest-resistant plant cultivars of *A. hybridus* along with aqueous neem leaf powder with neem plant bark ash extracts in an integrated management of pest insects; this combination is known to be environmentally safe, practicable, and sustainable. Research has also been conducted to combine varietal resistance with organic fertilizer application in reducing the infestation of cucumbers by insect pests [86]. The results suggest that fertilizer application may lead to increased crop productivity and greater plant vigour that may result in lowered susceptibility of crops to pests [86]. However, in some vegetable crops, application of fertilizer may intensify pest infestations as has been observed in cultivated cucumber [86].

## 5. Conclusions

Insect pests inflict damages to vegetable crops in Nigeria, and growers are aware of the menace of the vegetable pests and therefore apply control measures. There appears to be sufficient knowledge that can aid the formulation of effective and readily adoptable IPM packages for crop field insect pests of vegetable in Nigeria that may exclude the use of synthetic insecticides. There is no doubt that synthetic insecticide application is very effective against vegetable insect pests, but concerns for the environment and human health restrict their use globally. Proper cultural and agronomic practices for each vegetable crop, such as the choice of well-prepared fertile soil, planting pest-resistant vegetable varieties in intercropping systems and weed removal at the right times as reported by Muck [87] for IPM in rice, may be very relevant for vegetable crop IPMs. Ofuya [7] suggested an IPM system for cowpea in Nigeria with host plant resistance as the foundation to which any combination of, the manipulation of planting time, plant density and configuration, adequate weeding, intercropping and the use of aqueous formulations of plant-derived insecticides such as neem or the minimal application of proven synthetic insecticides. This suggestion may also be relevant in considering IPMs for vegetable crops. Clearly, the application of binary combinations of aqueous extracts of insecticidal plants as a component of any IPM package may significantly promote success in vegetable crop insect pest suppression. A binary combination of insecticidal botanicals in the IPM strategy may prevent or at least slow down the ability of insect pests to develop resistance since they become faced putatively with a more complex gamut of debilitating chemicals.

It is advantageous that many pesticidal plant extracts may improve yield and reduce insect pests on crops without harming beneficial arthropods [88]. By combining as many of these compatible IPM tools, the insect pest problems of vegetables in Nigeria can be reasonably mitigated in a sustainable manner. Any combination of IPM tools for any specific insect pest or group of pests of a particular vegetable and for specific locations should necessarily be subjected to empirical verification and appropriate recommendations made for the onward transmission to vegetable growers. The contemporary trend in the IPM concept is the inclusion of the management, profitability and sustainability aspects with emphasis on the importance of research and outreach [89]. Many areas for further research have been indicated in this review, such as the development of pest-resistant vegetable crop varieties, the use of entomopathogens, etc., which need the attention of researchers.

The new IPM paradigm requires cooperation among the gamut of scientists: soil scientists, agronomists, weed scientists, entomologists, plant pathologists, plant breeders, plant biotechnologists, agricultural economists, and agricultural extension experts in an interdisciplinary research approach to crop production constraints. Overall, amongst the IPM practices instituted for the mitigation of pest infestations on vegetable crops in Nigeria, the intercropping of suitable vegetables in combination with the application of aqueous extracts of *Azadirachta indica* and *Piper guineense* seeds under good farm hygiene and sanitation may prove most successful.

## Figures and Tables

**Table 1:** Major insect pests of some vegetable crops in Nigeria.

Vegetable Crop	Insect Pest			References
	Common Name	Scientific Name	Damage	
*Abelmoschus esculentus* (Okra)	Flea beetles	*Podagrica* spp.	Adults cause leaf holing	[8,14,15]
	Cotton Leafworm	*Spodoptera litoralis* Boisduval	Larvae feed on leaves leading to reduced yield	[8]
	Cotton Stainer Bug	*Dysdercus* sp.	Nymphs and adults attack fruits causing shrivelling and seed loss.	[8,14,15]
	Giant Coreid Bug	*Anoplocnemis curvipes* Fabricius	Nymphs and adults feed on new growth and developing fruits.	[8]
	Cotton Bollworm	*Helicoverpa armigera* Hubner	Larvae bore into buds and fruits, causing loss	[8]
*Solanum melogena*	Stem Borer	*Euzophera sharmotana* Rougeot	Larvae bore into fruits, causing loss.	[8]
(Eggplant)	Egg Fruit Borers	*Daraba laisalis* Walker	Larvae bore into fruits causing loss aided by pathogenic attack.	[8]
*Capsicum* spp. (Peppers)	Whitefly	*Bemisia tabaci* Gennadius	Transmits Pepper Veinal Mottle Virus (PVMV) that can reduce yield	[8]
	Peach-potato Aphid	*Myzus persicae* Sulzer	Insect feeding distorts young leaves and shoots. May transmit PVMV.	[8]
	Medfly	*Ceratitis capitata* Wiedermann	Larvae damage fruits and predispose them to fungal attack.	[8]
*Lycopersicon esculentum* (Tomato)	Fruit Borer	*H. armigera* Hubner	Larvae bore into fruits causing damage and predisposes them to fungal attack.	[8,14,15]
	Whitefly	*B. tabaci* Gennadius	Nymphs and adults feed on the underside of leaves, transmit viruses	[8,14,15]
	Cotton Leafworm	*S. litoralis* Biosduval	Larvae eat leaves, reducing leaf cover and yield	[8,14]
	Variegated grasshopper	*Zonocerus variegatus* Linnaeus	Defoliation by nymphs and adults	[8,15]
*Cucumis sativus* (Cucumber)	Crucifer flea beetle	*Phyllotreta cruciferae* Goeze	Defoliator	[26]
	Spotted Cucumber beetle	*Diabrotica undicempunctata* Howardi Barber	Defoliator	[26]
	Hadda Beetle	*Epilachna vigintiopunctata* Fabricius	Adults and larvae feed on plant leaves	[26]
	Skeletonizing Leaf Beetles	*Monolepta* spp.	Skeletonises leaves	[26]
*Citrullus lunatus* (Watermelon)	Pumpkin Flies	*Dacus bivitattus* (Bigot) *D. brevistylus* Bezzi	Larvae feed around the developing seeds in the fruit. Attack is usually followed by secondary rots.	[23]
*C. colocynthis* (Egusi melon)	Melon Fly	*Bactrocera cucurbitae* Coquillett	Larvae bore and eat into fruits, often destroying young fruits. Fruits are contaminated with frass and secondary attack by bacteria and fungi facilitated.	[8]
	Ladybird Beetle	*Epilachna* spp.	Adults and larvae attack and skeletonise leaves.	[8]
	Cotton Aphid	*Aphis gossypii* Glover	Adults and nymphs suck sap reducing growth and may transmit viruses.	[8]
*Amaranthus* spp. (Green Amaranths)	Green Stink Bug	*Nezara viridula* Linnaeus	Adults feed on flowers and seed heads, causing discoloration, shrivelling and premature drying of seeds, and reducing seed yield and viability	[8,14]
	Cotton Stainer Bug	*Dysdercus supersitiosus* Fabricius	Same as for Green Stink Bug	[8,14]
	Stink Bug	*Aspavia armigera* Fab.	Adults and nymphs suck leaf sap leaving clear spots on leaves	[8,15]
	Variegated grasshopper	*Z. variegatus* F.	Eats up foliage, reducing yield.	[8,14,15]
	Maize moth	*Spoladea recurvalis* F	Larvae feed on and skeletonise leaves, resulting in yield loss	[8]
	Leaf Webber	*Psara bipunctalis* Fabricius	Larvae cause webbing and damage to leaves, resulting in yield loss.	[8]
*Corchorus olitorius* (Jew’s Mallow)	Flea Beetles	*Podagrica* spp.	Adults attack leaves creating numerous small holes	[8]
	Leafworm	*Acraea* spp.	Larvae skeletonise leaves.	[8]
	Grasshoppers	Various species	Adults and nymphs feed on foliage.	[8]
*Hibiscus sabdariffa* (Roselle)	Whitefly	*B. tabaci* (Genn.)	Feed on leaf tissue and may transmit viruses	[16,30]
	Cotton Aphid	*A. gossypii* Glover	Adults and nymphs suck plant sap and may transmit viruses.	[16,30]
	Flea Beetles	*Podagrica* spp.	Leaf holing	[16,30]
	Spiny Bollworm	*Earias insulana* (Boisd.)	Larvae feed on swollen calyces and capsule wall of fruits.	[6,30]
	Leafhopper	*Empoasca* spp.	Xylem feeding insects and may transmit several destructive pathogens	[16,30]
*Vernonia amygdalina*	Bitterleaf weevil	*Lixus camerunus* Kolbe	Stem tunnelling and galling and lodging caused by larvae.	[14,15]
(Bitterleaf)				
	Grasshopper	*Zonocerus* spp.	Adults and nymphs feed on leaves.	[14,15]
	African mole cricket	*Gryllotalpa africana* Palisot de Beauvois	Feed on stems at and below ground level	[14]
	Coreid bug	*Anoplocnemis curvipes* Fab.	Adults and nymphs feed on young shoots	[29]
*Brassica oleracea* (Cabbage)	Cabbage Aphid	*Brevicoryne brassicae* L.	Adults and nymphs feed on sap and cause stunted and distorted growth; leaves disfigured with exuviae and sooty moulds.	[8]
	Variegated Grasshopper	*Z. variegatus* L.	Nymphs and adults defoliate plants	[8]
	Cotton Leafworm	*S. littoralis* Biosd.	Larvae defoliate plants	[8]
	Diamondback Moth	*Plutella xylostella* L.	Larvae defoliate plants	[8]
*Daucus carota* (Carrots)	Carrot root fly	*Psila* (*Chamaepsila*) *rosae* Fabricius	Holes in the centre of the carrot root. The outside of the carrot will have black blemishes, usually in rings around the carrot’s root.	[31]
	Aphids	Several species	Distort foliage, stunt growth and can kill young plants.	[31]
	Turnip moth	*Agrotis segetum* Denis & Schiffermuller	Larvae (caterpillars) damage edible roots.	[31]
*Allium cepa* (Onions)	Onion Thrips	*Thrips tabaci* Linderman	Adults and nymphs feed on plant sap, causing white spots on the leaves; infested plants may wilt and die.	[8,31]
	Cotton Leaf Worm	*S. litoralis* Biosd.	Larvae defoliate plants, causing a reduction in bulb size and weight.	[8]
	Crickets	Various species	Eat up bulbs	[8]
*Zingiber officinale* (Ginger)	Shoot Borer	*Conogethes punctiferalis* Guenee	Bores holes in pseudostem. Frass is thrown out of the holes and central shoot becomes yellow and withers.	[8]
	Rhizome Flies	*Colabata* spp.	The larvae bore into the rhizome and feed on it.	[27]
	Leaf Roller	*Udaspes folus* Cramer	Larvae feed and fold the leaves. Can result in complete defoliation.	[27]
	Rhizome Scale	*Aspidiella hartii* Cockerell	They suck sap from rhizomes and cause them to shrivel and dry up	[27]
*Curcuma longa*	Shoot Borer	*C. puntiferalis* Guen.	Larvae bore into rhizomes and feed on it.	[28]
(Turmeric)				
	Leaf Roller	*U. folus* Cramer	Larvae cause leaf folding and feed within.	[28]
	Yam Scale	*Aspidiella hartii* Cockerell	The scales damage the rhizomes both in field and storage.	[28]

**Table 2:** Vegetable crops and some recommended synthetic insecticides for insect pest control in Nigeria.

Vegetable Crop	Synthetic Insecticide	Target Insect Pest	References
Okra	Cypermethrin	All major insect pests	[8]
	Carbaryl	Flea beetles	[33,34]
Eggplant	Lambda-cyhalothrin or cypermethrin	Stem borer, Fruit Borer, Defoliators	[8,35,36]
Peppers	Lambda-cyhalothrin	Peach potato aphid, Fruit flies	[8]
Tomato	Permethrin, Lambda-cyhalothrin	Cotton Leafworm, Cotton Bollworm	[8]
	Cypermethrin	Tomato Fruit worm	[37,38]
Cucumber	Synthetic pyrethroids	Fruit Flies	[39,40]
Watermelon	Deltamethrin, Cypermethrin	Fruit Flies	[8]
Egusi Melon	Deltamethrin	Ladybird Beetles, Melon fly	[8]
	Cypermethrin, Fenitrothion, Dimethoate	Cotton Aphid	[8]
Green Amaranths	Pirimiphos-methyl	Green Stink Bug, Cotton Stainer, Leaf Caterpillars	[8]
	Carbaryl	Variegated Grasshopper	[8]
Jew’s Mallow	Deltamethrin	Flea Beetles, Leafworms	[8]
Cabbage	Carbofuran, Lamda-Cyhalothrin	Cabbage Aphid	[8,41]
Onions	Cypermethrin, Dimethoate	Onion Thrips	[8,42]
	Lambda-cyhalothrin, Endosulfan, Carbaryl	Cotton Leafworm	[8]

**Table 3:** Plants whose aqueous extracts can be sprayed to reduce insect pests of vegetables in Nigeria.

Plant Involved	Part Used	Vegetable Protected	References
*Azadirachta indica* A. Juss.	bark	*Amaranthus hybridus* L. *Celosia argentea* L. *Corchorus olitorius* L.	[60]
*Azadirachta indica* A. Juss.	seed oil	*Capsicum annum* L.	[61]
*Azadirachta indica* A. Juss.	Seed oil	*Citrullus lunatus* Thumb	[62]
*Azadirachta indica* A. Juss.	leaves	*Hibiscus sabdariffa* L.	[63]
*Azadirachta indica* A. Juss.	seeds	*Abelmoschus esculentus* L. Moench	[60,64]
*Azadirachta indica* A. Juss.	seeds	*Lycopersicon esculentum* L.	[37]
*Piper guineense* Schum and Thonn.	seeds	*Amaranthus hybridus* L. *Celosia argentea* L. *Corchorus olitorius* L.	[61]
*Piper guineense* Schum & Thonn	fruit	*Abelmoschus esculentus* L. Moench	[60,64]
*Ageratum conyzoides* L.	leaves	*Capsicum spp.*	[65]
*Ageratum conyzoides* L.	stem	*Abelmoschus esculentus* L. Moench	[66]
*Ocimum basilicum* L.	leaves	*Capsicum spp.*	[65]
*Senna hirsuta* (L.) Irwin & Barneby	leaves	*Capsicum spp.*	[65]
*Allium sativum* L	bulbs	*Capsicum annum* L.	[62]
*Allium sativum* L	bulbs	*Citrullus lunatus* Thumb	[63]
*Allium sativum* L	bulbs	*Abelmoschus esculentus* L. Moench	[67]
*Ricinus cummunis* L.	stem	*Abelmoschus esculentus* L. Moench	[66]
*Jatropha curcas* L.	leaves	*Abelmoschus esculentus* L. Moench	[66,68]
*Tephrosia vogelii* Hook.f.	leaves	*Hibiscus sabdariffa* L.	[30]
*Tephrosia vogelii* Hook.f.	leaves	*Abelmoschus esculentus* L. Moench	[69]
*Tephrosia vogelii* Hook.f.	leaves	*Cucumis sativus* L.	[40]
*Bridelia micrantha* (Hochst) Baill.	leaves	*Abelmoschus esculentus* L. Moench	[69]
*Dalbergia lactea* Vatke	leaves	*Abelmoschus esculentus* L. Moench	[69]
*Anonna squamosa* L.	leaves	*Abelmoschus esculentus* L. Moench	[70]
*Mitracarpus villosus* (L.)	leaves	*Lycopersicon esculentum* L.	[38]
*Balanites aegyptiaca* (Del.)	leaves	*Lycopersicon esculentum* L.	[38]
*Capsicum frutescens* L.	Fruits	*Abelmoschus esculentus* L. Moench	[64]
*Nicotiana tabacum* L.	Leaves	*Abelmoschus esculentus* L. Moench	[64]

## Data Availability

The copies of data and articles cited in the manuscript can be made available on request.
